# Prevalence of premenstrual syndrome and premenstrual dysphoric disorder among highly trained and elite female athletes: A systematic review and meta-analysis

**DOI:** 10.5114/biolsport.2025.148542

**Published:** 2025-03-18

**Authors:** Dominika Granda, Maria Karolina Szmidt, Olga Surała, Jadwiga Malczewska-Lenczowska

**Affiliations:** 1 Department of Nutrition Physiology, Institute of Sport, National Research Institute, 01-982 Warsaw, Poland; 2 Department of Human Nutrition, Institute of Human Nutrition Sciences, Warsaw University of Life Sciences (WULS-SGGW), 02-787 Warsaw, Poland

**Keywords:** Premenstrual syndrome, Premenstrual dysphoric disorder, Athletes, Sport, Menstrual cycle, Meta-analysis, Systematic review

## Abstract

Premenstrual syndrome (PMS) involves psychological and physical symptoms affecting around 30% of reproductive-age women, while premenstrual dysphoric disorder (PMDD) is a more severe, less common form. The aim of the review was to determine whether PMS and PMDD are more common among highly trained female athletes compared to non-training women.The study protocol was registered with PROSPERO (CRD42022323111). A search of PubMed, Scopus and Web of Science (up to August 2024) identified 12 eligible studies, with 7 included in the meta-analysis. Data from 1570 female athletes and 1165 non-athletes were analysed. A total of 755 cases of PMS and 54 cases of PMDD were found in all included studies. The prevalence of PMS among athletes ranged from 8.6 to 65.8%, while the impact of symptoms on athletic performance was reported by 41.1 to 44.3% of respondents. The meta-analysis on PMS prevalence (n = 2005 women) indicated 16% lower odds for athletes compared to non-athletes, however, the result was not statistically significant (p = 0.409). The prevalence of PMDD among athletes ranged from 1.3 to 13.1%. Meta-analysis results (n = 1314 women) showed 16% higher odds ratio of PMDD prevalence among athletes compared to non-athletes, but the association was also not significant (p = 0.660). This is the first systematic review and meta-analysis on the susceptibility of women to PMS and PMDD symptoms in relation to physical activity at a professional level. The prevalence of PMS among athletes could be highly variable, while PMDD appears to be a relatively minor issue. Further research regarding PMS and PMDD in female athletes is warranted.

## INTRODUCTION

The Paris Olympics of 2024 went down in history as the first Olympics with full gender parity on the field of play. This was possible thanks to the distribution of an equal number of quota places to female and male athletes by the International Olympic Committee (IOC). Despite this significant achievement in sports equality, there are still huge disparities in science-based knowledge. Most recommendations regarding optimal training and nutrition for athletes come from studies in which only men participated. Meanwhile, women have different physiology primarily due to hormonal differences, particularly in the context of the menstrual cycles [1, 2]. Therefore, using research results from male athletes may lead coaches to make inappropriate decisions and prevent women from achieving their maximum athletic potential [3]. There have not been many studies on the impact of the menstrual cycle on performance, and those that are available often have numerous methodological limitations. There are also few studies that have assessed the incidence and severity of menstrual disorders such as dysmenorrhea, menorrhagia or premenstrual syndrome (PMS). So far, only a few studies have been conducted on the occurrence of PMS in the population of training women, and even fewer among highly trained athletes. Also, few studies have assessed the impact of PMS on physical performance [4–7]. Moreover, most available research was conducted in small groups, mainly among Asian women.

Premenstrual syndrome refers to the psychological and physical symptoms that occur during the luteal phase of the menstrual cycle, significantly impacting women’s quality of life [8]. More than 200 symptoms have been described in the context of PMS, the most commonly reported ones including anxiety, irritability, breast tenderness, mood swings, and bloating [9]. A more severe form of PMS is premenstrual dysphoric disorder (PMDD) in which the same symptoms occur but with greater severity. PMDD symptoms also have a greater impact on the quality of life and may temporarily prevent normal functioning and lead to, for example, absence from work or training sessions [10]. The prevalence of PMS in the general population of women of reproductive age is estimated to be around 30%, while PMDD affects approximately 3–8%. However, the epidemiological data vary depending on where the study was carried out and due to the adoption of different diagnostic criteria. According to the results of a systematic review of the literature, the prevalence of PMS ranged from 40% in Europe to 85% in Africa [11]. Since the causes of PMS are unknown, available treatments are only symptomatic. There are pharmacological treatments such as selective serotonin reuptake inhibitors or oral contraceptives, while non-pharmacological treatments mainly involve lifestyle modifications in terms of diet, psychological therapy and physical activity [9].

Results on the association between recreational physical activity and PMS vary, depending on the type of study. Numerous interventional studies have demonstrated the effectiveness of regular physical activity (e.g., aerobic exercises, swimming, and yoga) in alleviating at least some symptoms of PMS [12–15]. However, findings from cross-sectional studies regarding the role of recreational physical activity in the risk of developing PMS are inconsistent: some indicate that higher levels of physical activity are associated with fewer symptoms [16, 17], while in others no such correlations were observed [18]. In a study by Morino et al. [19], a U-shaped relationship was identified: women engaging in moderate physical activity were the least likely to experience premenstrual symptoms, whereas the risk was elevated among those with both high and low activity levels, with the highest risk observed in women with low activity [19]. At the other extreme of this U-shaped relationship are women who train competitively at a professional level. Professionally practised sport involves not only significant physical exertion, but also substantial stress, stemming from competition, performance pressure, high expectations of good results, and, in many cases, financial challenges [20]. However, there is a limited amount of research examining the association between elite-level physical activity and the occurrence of PMS and PMDD.

**FIG. 1:**
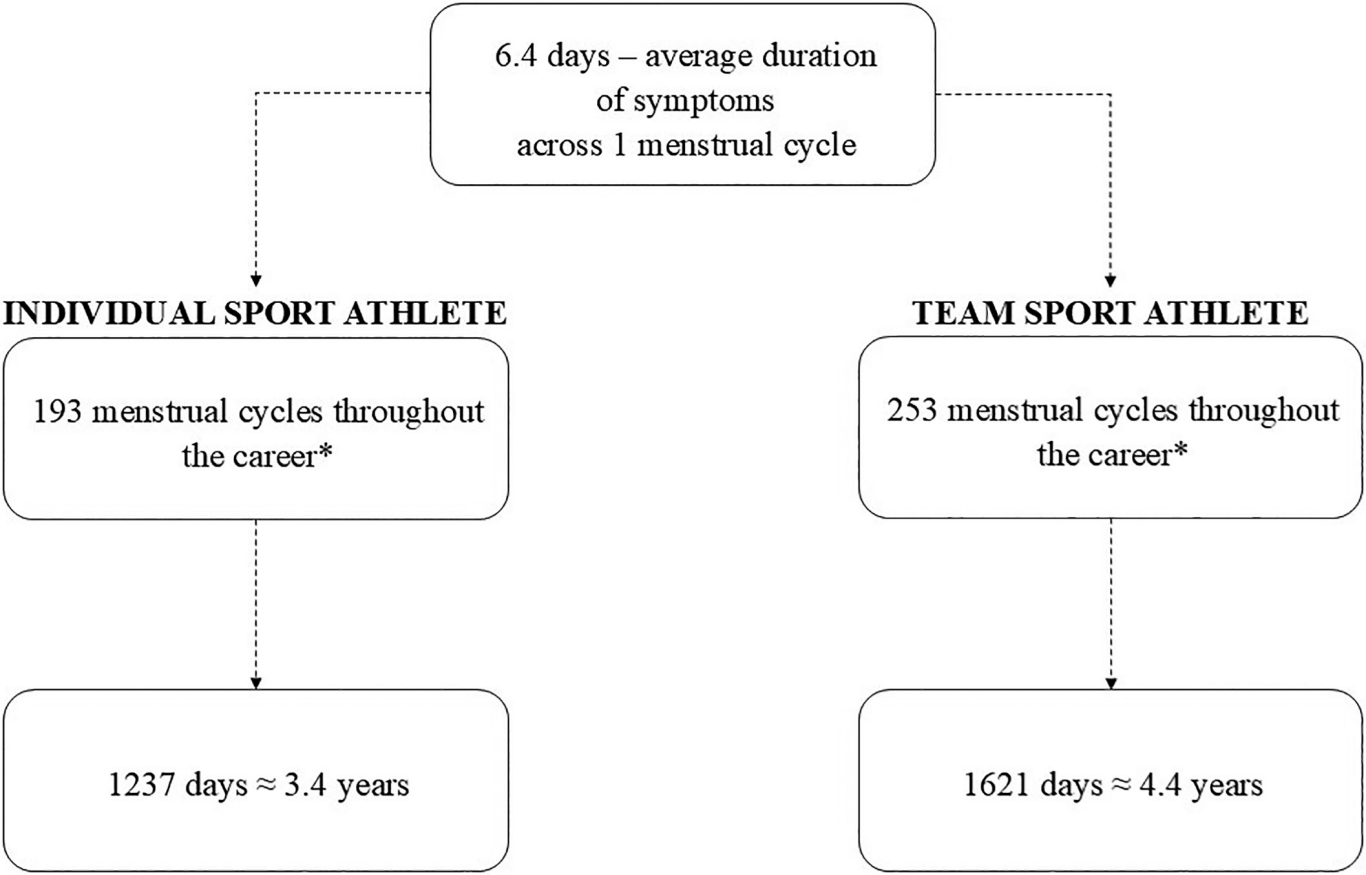
The potential impact of premenstrual symptoms on a career of individual and team sport athlete. *assuming that the median menarche age is 11.9 years old, and the age of sport retirement for individual athlete is 28 years old, and for team sport athlete 32.

Assuming that the median menarche age is 11.9 [21], and that individual sport athletes usually retire at the age of 28, while elite team athletes retire at the age of 32 [22], the total length of the impact of PMS on female athletes can be estimated. In a healthy female, the average menstrual cycle number per year is 13 [23], and average length of premenstrual symptoms occurrence is 6.4 days [24]. Consequently, female athletes may experience symptoms for 1237–1621 training days during their career (Figure 1). This is a total of 3–4 years of symptoms, which, if severe, may significantly affect well-being but also sports performance, competition readiness, and according to some reports, also the risk of injury [4]. Due to the diversity of research methods and inconsistent results, the need to structure the currently available data was noted. Therefore, the aims of this systematic review and meta-analysis were as follows:

1. To review available studies on PMS and PMDD prevalence in highly trained and elite female athletes.
2. To estimate the mean effect size of physical activity on the prevalence of PMS and PMDD in highly trained female athletes.

## MATERIALS AND METHODS

The protocol for this review has been registered in the International Prospective Register of Systematic Reviews (CRD42022323111). The current systematic review was based on the Preferred Reporting Items for Systematic Reviews and Meta-Analyses (PRISMA) guidelines.

### Literature search

Selected databases (Web of Science, PubMed, Scopus) were searched by two authors independently (DG and OS). The initial systematic review was conducted in March 2022, with an update in January 2023 and August 2024. For searching the following key terms and the conjunctive normal form were used: ((premenstrual syndrome) OR (premenstrual dysphoric disorder) OR (PMS) OR (PMDD)) AND ((athletes) OR (sport) OR (athletics) OR (athlete)). Additional manual search of the retrieved articles was performed to identify other potentially eligible studies. In the case of meta-analysis, one additional criterion was used: the occurrence of PMS and/or PMDD in the group of athletes had to be compared to the occurrence in the group of non-athletes.

### Inclusion criteria

In brief, for the systematic review we included studies with the participation of highly trained athletes at least at the national level (participating in Provincial/State or Academy Programmes), who engaged in regular exercise training [25, 26]. Other inclusion criteria were as follows: (1) assessment of PMS or/and PMDD with a properly described method, (2) cross-sectional, case-control, or prospective study design, (3) publications written in English, (4) presenting in the results the prevalence of PMS or/and PMDD in athletes. An additional inclusion criterion for the meta-analysis was the presence of a comparison of the prevalence of PMS and/or PMDD between groups of female athletes and non-athletes. The main exclusion criteria were: (1) interventional study design, (2) no full-text access, (3) review studies, (4) no information on level of physical activity. The above criteria were applied by two authors (DG, OS) while deciding whether to include an article in the review based on its abstract or full text, and in case of a disagreement, a consensus was achieved through discussion with other authors (JML, MKS).

### Data extraction and analysis

All studies included in the review were analyzed independently by two authors (DG, OS). The data were entered into appropriate data tables, separate for the study characteristics and separate for the main results. In terms of study characteristics, data concerning authors’ names, year of the publication, country, study design, numbers of athlete and non-athlete group (if present), mean age in athlete and non-athlete groups (if present), sport discipline, % of elite athletes, and PMS assessment method were obtained. In terms of the main results, data on PMS and/or PMDD prevalence in the athlete and non-athlete group with the p-value were obtained. Where possible, data on the impact of symptoms on sports performance were also collected.

### Quality assessment

The quality of the studies was assessed independently by two authors (DG, MKS) according to a modification of the Newcastle–Ottawa Quality Assessment Scale (NOS) for cross-sectional studies [27]. Each study was assessed in terms of 3 categories: selection, comparability, and outcome. The maximum of total points was 10; 0 to 3 points indicated a low-quality study, 4 to 6 points a medium-quality study, and 7 to 10 points a high-quality study. The results of quality assessment are presented in Table 1. Details are described in the Supplementary Materials.

**TABLE 1:** Quality assessment of studies included in the systematic review using a modified Newcastle–Ottawa Quality Assessment Scale for cross-sectional studies [27]

Authors	Selection (Max. 5 points)	Comparability (Max. 2 points)	Outcome (Max. 3 points)	Sum (Max. 10 points)	Quality
Czajkowska et al. 2015 [36]	2	2	3	7	High
Czajkowska et al. 2019 [35]	2	0	3	5	Medium
Fekr et al. 2012 [34]	1	1	1	3	Low
Foster et al. 2019 [38]	2	2	3	7	High
Gaion and Vieira, 2011 [39]	3	1	2	6	Medium
Hasdemir et al. 2016 [40]	2	2	2	6	Medium
Mukherjee et al. 2014 [41]	2	2	0	4	Medium
Takeda et al. 2015 [6]	1	1	1	3	Low
Takeda et al. 2016a [4]	3	1	2	6	Medium
Takeda et al. 2016b [5]	1	1	1	3	Low
Yamada et al. 2018 [7]	1	1	1	3	Low
Yi and Bae, 2021 [37]	2	2	3	7	High

Range of total points: 0–10: 0 to 3 points—low-quality study, 4 to 6 points—medium-quality study, and 7 to 10 points—high-quality study

### Meta-analysis

Meta-analysis was performed by one of the authors (MKS) using Comprehensive Meta-Analysis Version 4 [28]. Given that significant variability in methods and populations was expected between studies, a random-effects model was used in the analyses [29], which assumes that the true effect varies from study to study [30] and that studies in the meta-analysis are a random sample from a universe of potential studies [28, 30–33]. The calculated effect size index was the odds ratio.

## RESULTS

**FIG. 2:**
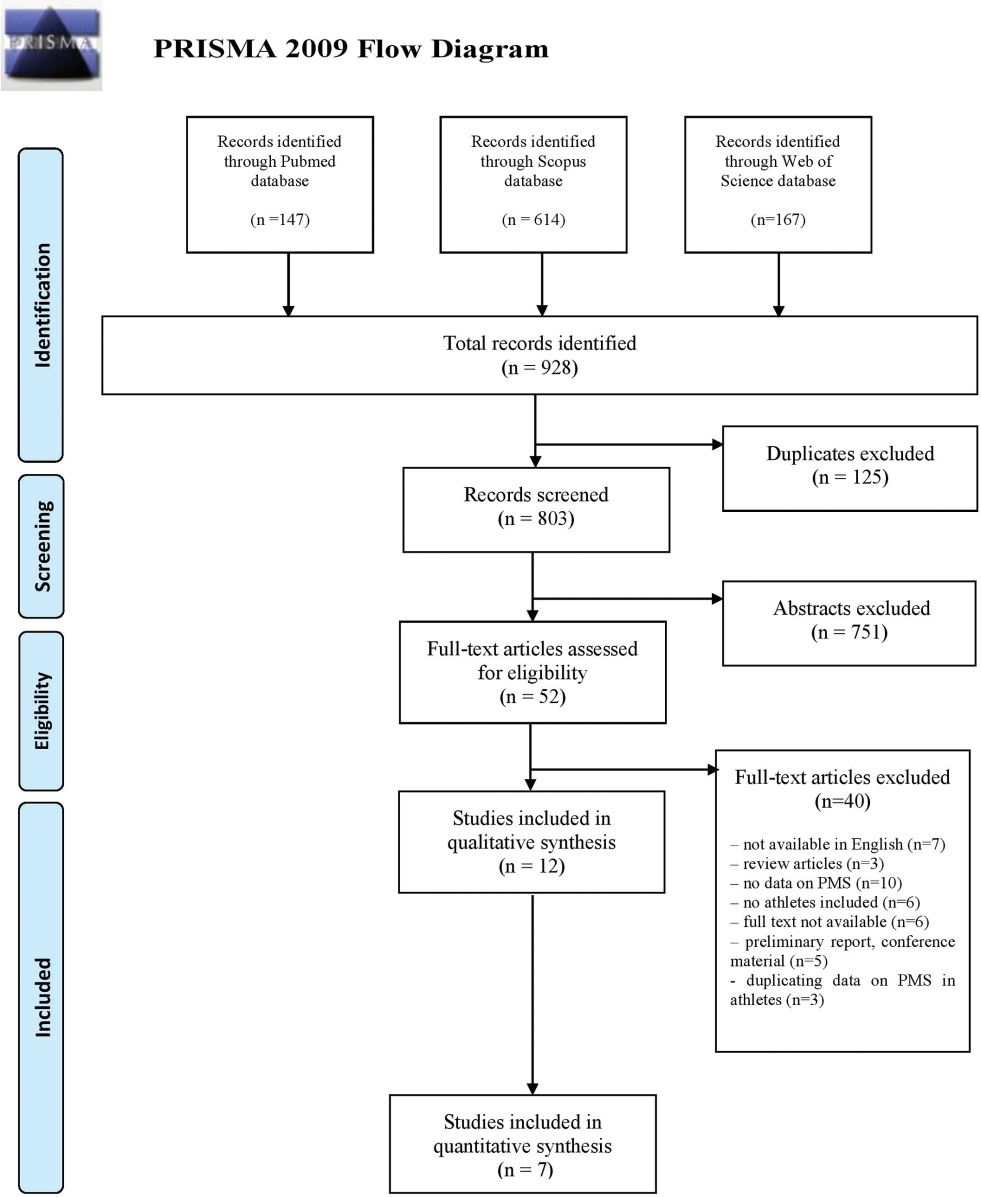
Literature review and meta-analysis flow diagram of the selection process according to the Preferred Reporting Items for Systematic Reviews and Meta-Analyses (PRISMA) Statement.

Nine hundred and twenty-eight papers were identified in the initial database search (147 from PubMed, 614 from Scopus and 167 from Web of Science). The selection of papers for this review was performed according to the PRISMA flow chart (Figure 2). In accordance with the adopted inclusion criteria, 12 studies qualified for the systematic review, and 7 of them were included in the meta-analysis. The characteristics of the included studies are summarized in Table 2. The eligible studies were published between 2011 and 2021. Most studies were conducted in Asia, including 4 in Japan and one each in South Korea, Iran and India. Three of the studies included in the review were conducted in Europe, including two in Poland and one in Turkey. Two studies from South America were carried out in Brazil. All studies based on obtained data had cross-sectional character. The sizes of groups ranged from 25 to 394 female athletes and from 40 to 618 non-athlete-females. In total, data from 1570 female athletes and 1165 non-athletes were included in the systematic review. The diversity of the studied disciplines was substantial; only two studies were limited to the assessment of PMS in a specific discipline, while the remaining studies examined female athletes practising various sports. The tools used to assess PMS and PMDD were vastly diverse. In 3 of the 12 studies the validated Premenstrual Symptoms Screening Tool (PSST) was used, in 4 others the twin Premenstrual Symptoms Questionnaire (PSQ) scale was used, in another 3 an original questionnaire based on the PMS diagnostic criteria presented by the American College of Obstetricians and Gynecologists’ guidelines. Fekr et al. [34] were the only ones to choose the Premenstrual Daily Symptom Diary (PDSD) scale. Data on the occurrence of PMS and PMDD in the included studies are presented in Table 3. The assessment of the methodological quality of the studies included in the review revealed that 41.7% of the articles were of medium quality, 33.3% were of low quality, and the remaining 25.0% were of high quality (Table 1). It is worth pointing out that all the studies categorised as high quality scored 7 out of 10, which was also the highest score.

**TABLE 2:** Characteristics of the studies included in the systematic review and partially in the meta-analysis

Author, year	Country	Study design	Number of athletes	Number of non-athletes	Age of athletes	Age of non-athletes	Sport discipline	% of elite athletes	PMS questionnaire
Czajkowska et al. 2015 [36]	Poland	CS	75	50	18.7 ± 1.9 (16–22)	18.5 ± 1.8 (16–22)	Competitive sports	–	PSST, AC

Czajkowska et al. 2019 [35]	Poland	CS	45	40	16.3 ± 0.8 (15–17)	16.9 ± 1.0 (15–17)	Rhytmic Gymnastic	–	PSST, AC

Fekr et al. 2012 [34]	Iran	CS	180	180	21.4 ± 4.3	20.6 ± 2.1	Various (not mentioned)	–	PDSD

Foster et al. 2019 [38]	Brazil	CS	52	–	19.8 ± 4.7	–	Soccer	–	DSR

Gaion and Vieira, 2011 [39]	Brazil	CS	25	–	18–49	–	Handball, basketball, karate, track and field, others	–	AC

Hasdemir et al. 2016 [40]	Turkey	CS	87	85	20	18	Gymnastics, folk dancing, volleyball, others	–	AC

Mukherjee et al. 2014 [41]	India	CS	79	80	17.2 ± 1.4	17.0 ± 1.3	Various track and field sports	–	AC

Takeda et al. 2015 [6]	Japan	CS	174	618	20.2 ± 1.1 (18–23)	16.7 ± 0.9 (15–19)	Fighting sports, ball games, archery, swimming, track, and others	66.7%	PSQ

Takeda et al. 2016a [4]	Japan	CS	394	–	16.4± 0.78	–	Ball games, fighting sports, track, gymnastics, swimming, others	–	PSQ

Takeda et al. 2016b [5]	Japan	CS	200	112	19.7 ± 1.26*		Ball games, fighting sports, archery, swimming, triathlon, yachting	–	PSQ

Yamada and Takeda, 2018 [7]	Japan	CS	135	–	18–23	–	Combat or martial arts, archery, ball sports, athletics, others	–	PSQ

Yi and Bae, 2021 [37]	South Korea	CS	124	–	24.8 ± 4.3	24.3 ± 4.8	Field hockey, softball, sepak takraw, artistic swimming, wrestling, weight lifting, others	100%	PSST

* the authors provided only average age for all the participants combined, AC – author’s questionnaire, CS – cross-sectional, DSR – Daily Symptom Record, MDQ – Menstrual Distress Questionnaire, NA – data not available, PDSD – Premenstrual Daily Symptom Diary, PSQ – Premenstrual Syndrome Questionnaire, PSST – Premenstrual Symptoms Screening Tool

**TABLE 3:** Results of the studies included in the systematic review and partially in the meta-analysis

Author, year	Prevalence of PMS, % (n)			Prevalence of PMDD, % (n)		
	athletes	non-athletes	p-value	athletes	non-athletes	p-value
Czajkowska et al. 2015 [36]	49.33 (37)	32.0 (16)	0.045	9.3 (7)	6.00 (3)	p > 0.05
Czajkowska et al. 2019 [35]	48.9 (22)	32.5 (13)	0.12	13.1 (6)	5.00 (2)	0.19
Fekr et al. 2012 [34]	55.9 (106)	66.1 (119)	0.157	–	–	–
Foster et al. 2019 [38]	59.6 (31)	–	–	–	–	–
Gaion and Vieira 2011 [39]	48.0 (12)	–	–	–	–	–
Hasdemir et al. 2016 [40]	36.8 (32)	54.1 (46)	0.02	–	–	–
Mukherjee et al. 2014 [41]	65.8 (52)	85.0 (68)	0.01	–	–	–
Takeda et al. 2015 [6]	8.6 (15)	11.8 (73)	p > 0.05	2.9 (5)	2.6 (16)	p > 0.05
Takeda et al. 2016a [4]	8.9 (35)	–	–	1.3 (5)	–	–
Takeda et al. 2016b [5]	14.5 (29)	14.3 (16)	0.652	2.5 (5)	4.5 (5)	0.652
Yamada and Takeda, 2018 [7]	11.1 (15)	–	–	–	–	–
Yi and Bae, 2021 [37]	14.5 (18)	–	–	–	–	–

PMDD – Premenstrual Dysphoric Disorder; PMS – Premenstrual Syndrome

### Results of the systematic review

#### PMS prevalence in athletes

A total of 755 cases of PMS were found in all included studies, including 404 in athletes (25.7%) and 351 in non-athletes (30.1%). Based on all 12 included studies [4–6, 34–41], the prevalence of PMS in the group of athletes ranged from 8.6 to 65.8%. In seven studies the percentage of women with PMS in the group of athletes was compared to that in the group of non-athletes. Statistically significant differences between groups were observed in only 3 studies [36, 40, 41], including 2 medium quality studies. PMS occurred significantly less frequently in the group of athletes [40, 41], and in one high quality study it occurred significantly more often [36].

### PMDD prevalence in athletes

A total of 54 cases of PMDD were found in all included studies, including 28 in athletes (3.2%) and 26 in non-athletes (3.2%). Based on the results of five studies [4–6, 35, 36] which differentiate PMDD from PMS, PMDD prevalence among athletes varied from 1.3 [4] to 13.1% [35]. Yi and Bae (2021) [37] mentioned 1 athlete who met the PSST criteria for PMDD diagnosis; however, due to the low percentage of this condition in the studied group of athletes, they decided not to separate it and treated PMS and PMDD together. In four [5, 6, 35, 36] out of five studies the authors compared PMDD prevalence in athletes to its prevalence in a non-athlete population; no statistically significant differences were observed (p > 0.05).

### Impact of PMS and PMDD on athletic performance

In three studies, Takeda et al. [4–6] asked female athletes to determine whether and to what extent PMS symptoms affected their athletic performance during training or competition (Table S1 in Supplementary Materials). The impact of symptoms on athletic performance was reported by 41.1 to 44.3% of respondents. In most cases the symptoms affected them in a mild way (28.2 to 31.6%), less often in a moderate way (8.0 to 11.5%). A severe impact was reported by 2.3 to 4.6% of the surveyed athletes.

### Results of the meta-analysis

#### PMS prevalence in athletes

**FIG. 3:**
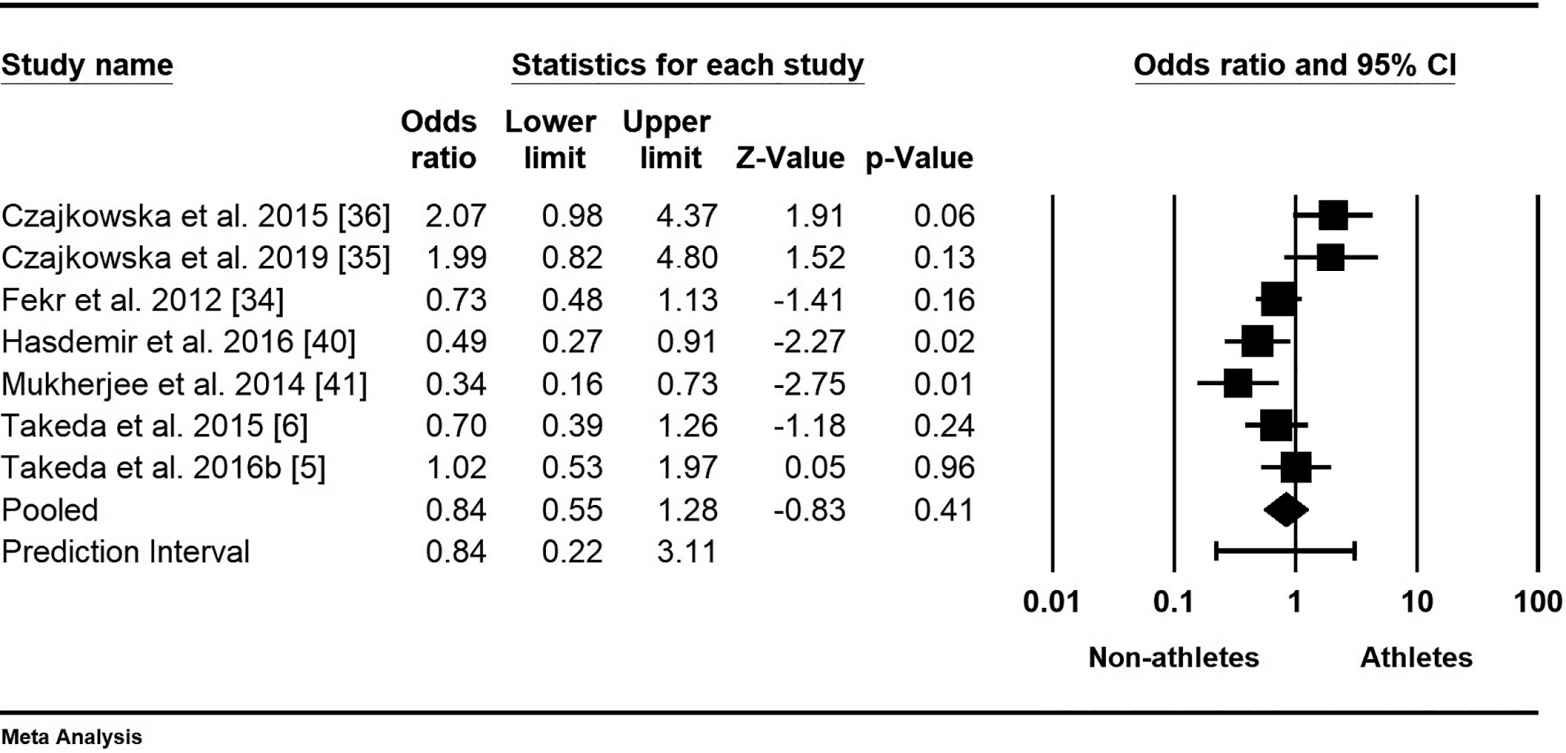
Forest plot of odds ratios (ORs) and 95% confidence intervals (CIs) for premenstrual syndrome (PMS) among female athletes and non-athletes. The black squares represent the ORs for each study, with their sizes reflecting the weight of the respective studies in the meta-analysis. The horizontal lines indicate the corresponding 95% CIs. The diamond at the bottom center represents the overall summary estimate of the OR, with its width denoting the 95% CI.

The meta-analysis on the prevalence of PMS in female athletes was based on seven studies with a total of 2005 participants [5, 6, 34–36, 40, 41]. The mean odds ratio was 0.84, indicating lower odds for non-athletes, with a 95% confidence interval of 0.55 to 1.28 (Figure 3). The null hypothesis, stating that the mean effect size is 1.00, could not be rejected (Z-value = -0.83, p = 0.409). However, the null hypothesis that all studies in the analysis shared a common true effect size was rejected, as indicated by a Q-value of 18.30 with 6 degrees of freedom (p = 0.006). The I-squared statistic showed that 67% of the variance in observed effects reflects true effects rather than sampling error. Additionally, the results indicated that the true effect size in 95% of all comparable populations falls within the interval of 0.22 to 3.11, while the variance of true effect sizes (tau-squared) is 0.22 in log units.

### PMDD prevalence in athletes

**FIG. 4:**
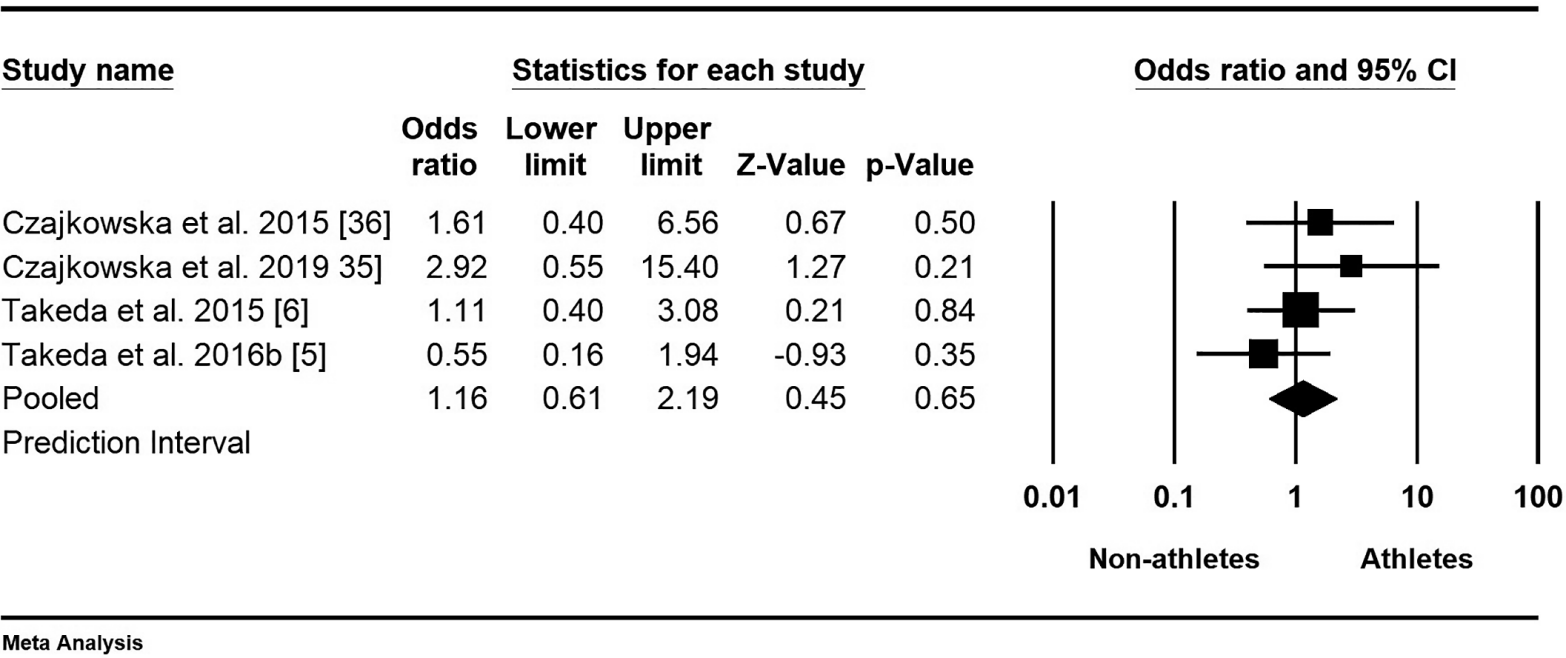
Forest plot of odds ratios (ORs) and 95% confidence intervals (CIs) for premenstrual dysphoric disorder (PMDD) among female athletes and non-athletes. The black squares represent the ORs for each study, with their sizes reflecting the weight of the respective studies in the meta-analysis. The horizontal lines indicate the corresponding 95% CIs. The diamond at the bottom center represents the overall summary estimate of the OR, with its width denoting the 95% CI.

Data on the prevalence of PMDD in athletes were available from four studies with a total of 1314 participants [5, 6, 35, 36]. The mean odds ratio was 1.16, indicating higher odds for athletes, with a 95% confidence interval of 0.61 to 2.19 (Figure 4). This means that in the universe of comparable studies, the effect size could fall anywhere within this interval; however, the null hypothesis, stating that the mean effect size is 1.000, could not be rejected (Z-value = 0.45, p = 0.66). The results showed that all studies shared a common effect size (tau-squared = 0), indicating no dispersion of true effects. Therefore, we did not report a prediction interval.

## DISCUSSION

To our knowledge, this is the first systematic review combined with a meta-analysis in which the incidence of PMS and PMDD was compared in highly trained female athletes and the general population of women in reproductive age. The prevalence of PMS among female athletes ranged from 8.6 to 65.8%, while the impact of symptoms on athletic performance was reported by 41.1 to 44.3% of respondents. The prevalence of PMDD among athletes ranged from 1.3 to 13.1%. Meta-analysis results of PMS (n = 2005 women) and PMDD (n = 1314 women) prevalence showed no significant difference in odds ratio between female athletes and non-athletes (p = 0.66 and p = 0.409, respectively). As demonstrated in this systematic review, the research findings are too inconsistent to draw a clear conclusion on whether highly trained female athletes are more or less prone to experiencing PMS compared to non-athletes. These results are in line with those of Karal et al. [42], who also did not find significant a difference in the occurrence of PMS between athletes and sedentary students. The prevalence of PMS in female athletes varied widely between studies included in this review, ranging from 8.6% [6] to 65.8% [41]. According to our results, the lowest percentage of PMS cases was reported in studies involving Japanese female athletes [4–7]. This is consistent with previous research indicating that the prevalence of PMS in the general population of Japanese women is lower than in the population of women living in Western countries [43], which may be due to cultural factors and a low-fat diet [43]. In another, independently conducted systematic review assessing the occurrence of menstrual cycle disorders and menstrual cycle-related symptoms [44], the occurrence of PMS and PMDD in a population of female athletes was similar to that in this review. It should be noted that Taim et al. [44] included only 4 studies on PMS and 5 studies on PMDD, whereas in this review, the same studies on PMDD were considered, but 11 studies on PMS were included (only 3 of which overlapped with those considered by Taim et al. [44]). Taim et al. [44] however did not conduct a meta-analysis. The present meta-analysis indicated a mean 16% lower odds of PMS among non-athletes; however, this result was not statistically significant. Additionally, the prediction interval of 0.22 to 3.11 suggests that further investigation is necessary to draw definitive conclusions regarding the prevalence of PMS in athletic populations. In the case of PMDD, the mean odds ratio also showed 16% higher odds in athletes compared to non-athletes, but this finding was not statistically significant either. Furthermore, the prediction interval could not be calculated. This indicates that while there may be a tendency for increased prevalence of PMDD in female athletes, we cannot conclusively state that they are more likely to experience PMDD than non-athletes, and further research on this topic is needed.

Although the review did not find that female athletes had a higher risk of PMS than the general population, this does not rule out the possibility that they may be more predisposed to experiencing some PMS symptoms than non-training women of reproductive age. The results of some other studies provided supporting evidence for this. For example, Takeda et al. [6] reported that the severity of anxiety/tension, anger/irritability, decreased interest in work, home, or social activities, overeating or food cravings and physical symptoms was statistically significantly higher in athletes than in a non-athlete group which consisted of high school students. Sport-related performance anxiety and various disorders on the spectrum from disordered eating to eating disorders are well-known issues, and are especially prevalent among female athletes [45]. Further research should clarify whether these disorders are exacerbated by the menstrual cycle. Czajkowska et al. [36] showed that breast swelling and tenderness was significantly more prevalent among athletes than non-athletes (69.33% vs. 48.0%, p = 0.02, respectively). Even though exercise-induced breast pain and premenstrual mastalgia are long-standing issues, the research in this area among female athletes is limited. In a study conducted among 540 Australian female athletes, 63% of the participants reported experiencing breast pain associated with the menstrual cycle, and 33% indicated that these symptoms worsened during physical activity. Additionally, 43% of the participants reported breast pain unrelated to the menstrual cycle but linked to physical activity itself. In both cases, the symptoms significantly impacted sports performance in 20% and 30% of the participants, respectively [46].

### PMS and PMDD in elite athletes: a persisting knowledge gap

**FIG. 5:**
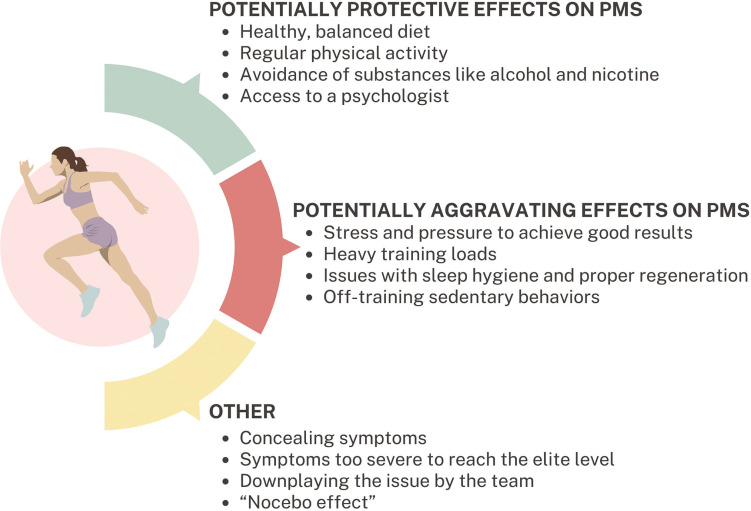
Factors with potential protective and aggravating effects on the occurrence of premenstrual symptoms in elite female athletes, as well as key considerations for designing and analysing studies on premenstrual syndrome (PMS) and premenstrual dysphoric disorder (PMDD) in this population.

An important problem is the low availability of studies on PMS and PMDD with the participation of elite athletes – this review included only 1 study concerning solely elite athletes [37], and one in which they constituted the majority of the subjects (66.6%) [6]. Takeda et al. [6] found that elite athletes had significantly higher risk of poor athletic performance due to premenstrual symptoms: OR 8.63 (95%CI: 1.22–120.0). Elite athletes are exposed to greater stress and pressure to perform well than lower-level athletes, which could predispose them to a greater extent to PMS and PMDD. However, in the two studies involving elite athletes included in this review [6, 37], the percentage of women with PMS was relatively low. The explanation for the low percentage of women with different premenstrual symptoms in the group of highly trained athletes could be that the symptoms may be so severe that they prevented them from pursuing a high-level sports career. However, this hypothesis cannot be confirmed by research. Additionally, professional athletes typically pay attention to their diet, sleep hygiene, and other lifestyle factors to optimize athletic performance. Although the etiology of PMS is not fully understood, research results indicate that an improper diet rich in processed products, sugar and sweets [47, 48], as well as a sedentary lifestyle [16, 17], contribute to the occurrence of PMS symptoms. Therefore, conscientiousness regarding these lifestyle elements in elite female athletes [49] may also explain the relatively low rates of PMS and PMDD. It is also important to note that with the increase in the level of competition, there is a corresponding rise in sports funding and access to a team of specialists, such as a dietitian, psychologist, or physician. The opportunity to consult professionals regarding symptoms, coupled with a sense of support, may contribute to a reduction in symptom severity. Athletes competing at lower levels may face similar training loads and exert comparable effort, yet they may not receive adequate support from a team of specialists. Furthermore, intensive training at sub-elite levels often necessitates balancing long training sessions with professional obligations outside of sports, and in many cases, with family responsibilities. This combination can induce stress and elevate the risk of PMS and PMDD [50]. In the group of elite athletes (though this probably also applies to lower-level athletes), the reluctance to disclose the symptoms may also be a limitation. Female athletes may perceive PMS symptoms as a weakness or excuse with which they do not want to be associated [6]. This may result from their own beliefs, or the beliefs of the coach or other staff members such as a physician or physiotherapist. Coaches’ knowledge of the menstrual cycle and its potential impact on performance is insufficient, especially among men. This is supported by the results of a study from Japan, showing that 30% of male coaches training women did not know what PMS was [51]. Another important factor to consider when planning research on PMS in female athletes is the nocebo effect (NE), which may provide a possible explanation for the self-awareness of PMS symptoms [52]. NE arises from negative expectations that can be triggered through verbal suggestions, conditioning, or social observation, significantly influencing the perception of PMS symptoms. Female athletes, more often than non-athletes, undergo assessments of body parameters such as performance, weight, or body composition. Such frequent measurements may contribute to increased body awareness and their changes, including those related to the menstrual cycle [53–55]. This careful observation of their bodies and experienced symptoms, such as weight gain or bloating, may predispose athletes to be more attuned to noticing and experiencing other PMS symptoms [56, 57]. Due to the limited availability of studies involving elite athletes, it was impossible to conduct a meta-analysis including only professional athletes at the highest level. Various factors related to elite athletes that may have both positive and negative effects on the risk of experiencing PMS within this group are illustrated in Figure 5. The figure also highlights additional variables that should be taken into account in future research on this subject: these issues will be discussed in the subsection “Implications for further research”.

### Potential mechanism of PMS and PMDD occurrence in female athletes

The aetiology of PMS is unknown, although several hypotheses have been proposed to explain the potential mechanism of symptom development. Given the cyclical nature of the symptoms and their close correlation with the phases of the menstrual cycle, it was hypothesized that they were linked to hormonal imbalances within the hypothalamic-pituitary-ovarian axis. However, no differences were found in hormone levels in women with PMS and healthy women, regardless of the cycle phase [58]. It is currently believed that women with PMS are hypersensitive to changes in hormone levels that occur physiologically during the menstrual cycle, but this has not been directly demonstrated in studies [59]. Another hypothesis posits the involvement of allopregnanolone, a strong agonist of the γ-aminobutyric acid (GABA) A receptor, in the modulation of the inflammatory process [60, 61]. In healthy women, allopregnanolone, by binding to the GABA A receptor, reduces inflammatory processes in the body and has an anti-anxiety effect. It is presumed that in women with PMS, allopregnanolone has the opposite effect, thereby contributing to the development of symptoms [60, 62]. An alternative theory suggests that serotonin plays an important role in the development of PMS symptoms. There is evidence indicating reduced serotonin levels in the luteal phase, lower binding of serotonin to its transporter on platelets, and increased symptoms of premenstrual irritation after the depletion of tryptophan stores in women with PMS [61, 63]. Oestrogens may also influence mood and behaviour through the serotonergic system [64]. On the other hand, previous studies [65] demonstrated that severe stress may intensify the symptoms of PMS and PMDD, which may be the result of stress-induced alterations in the neuroendocrine system. According to yet another hypothesis, PMS symptoms are the result of a number of neuroendocrine reactions triggered by ovulation, rather than simply a change in the sex hormones. Changes in behaviour and emotions during PMS are attributed to the neuron functions and trophic factors involved in signalling and synaptic integration. The most important neurotrophin in this context is considered to be brain-derived neurotrophic factor (BDNF) [66]. A decreased BDNF level in serum was observed in people with mood and eating disorders and depression [67]. Simultaneously, BDNF appears to be the primary neurotrophin involved in sports performance, with strong evidence indicating that acute exercise serves as a stimulus for its elevation [68, 69]. Its variability in the menstrual cycle was also found: in healthy, naturally menstruating women, the concentration of BDNF in the luteal phase was up to three times higher than in the follicular phase [70]. Unfortunately, to date there have been no studies evaluating BDNF concentrations in elite female athletes in the context of PMS with respect to menstrual cycle phase.

### Strengths and limitations

To the best of our knowledge, this is the first systematic review and meta-analysis to compare the incidence of PMS and PMDD between highly trained female athletes and the general population of women of reproductive age. The main strength of this review lies in its strict adherence to the PRISMA guidelines [71] for conducting a systematic review. Two independent researchers conducted the database searches and pre-selected publications for inclusion, and the quality assessment of the studies was also performed independently by both researchers. The inclusion and separate analysis of PMS and PMDD is also a strength of the study.

The limitations of this systematic-review and meta-analysis include the small number of studies available for inclusion and the variations in methodologies across those studies. Due to the limited number of studies and small study groups, it was not possible to analyse sub-groups by age or sport discipline, for example. Furthermore, regarding meta-analysis, the general rule is that estimates of heterogeneity based on less than ten studies are not likely to be reliable, which underscores the need for further research on this topic.

### Implications for further research

Determining the prevalence of PMS and PMDD among highly trained female athletes is significant for several reasons. First, the symptoms of PMS and PMDD can substantially impact sports achievements, potentially affecting up to 1,500 days over the course of their career path. Second, awareness of this issue among coaches and female athletes themselves remains insufficient, even though studies have shown that educational interventions can yield measurable benefits [72]. Assessing the scale of the problem could serve as a starting point for designing educational initiatives and planning interventional studies involving female athletes. When conducting studies on the prevalence of PMS and PMDD in the athletic population, the choice of assessment tools is critical. The studies included in this review reveal considerable variability in the methods used, complicating crossstudy comparisons. The optimal approach involves employing validated, prospective tools (e.g., the Daily Symptom Rating Scale), although validated retrospective methods, such as the Premenstrual Symptoms Screening Tool, are also acceptable. Additionally, it is essential to evaluate the level of sports competition and current training loads to objectively define the impact of training and performance caliber.

One particularly under-researched group is that of elite female athletes. Research in this population, however, must consider factors that may influence the study outcomes. For instance, some athletes may be reluctant to disclose symptoms for fear that doing so might be perceived as a weakness. Due to the low level of awareness among coaches and team staff, athletes may also worry about how their symptoms will be perceived by their team or fear that their concerns will be dismissed. Conversely, some athletes might experience a nocebo effect, arising from frequent testing and increased monitoring of their bodily symptoms. It should also be noted that accurately determining the prevalence of PMS and PMDD among elite athletes might be inherently challenging, as severe symptoms may have precluded some individuals from reaching this level of athletic achievement.

## CONCLUSIONS

This is the first systematic review and meta-analysis on the susceptibility of women to PMS and PMDD symptoms in relation to physical activity at a professional level. The prevalence of PMS among athletes may vary widely, while PMDD appears to be a relatively small issue, perhaps because its severity may prevent women from pursuing a sports career. Nevertheless, this remains a matter of speculation and is difficult to substantiate through research. Furthermore, based on general estimation derived from the reviewed articles, there is a slightly higher prevalence of PMS among non-athletes compared to athletes, while the prevalence of PMDD appears similar in both groups. This potential trend was not supported by meta-analysis, possibly due to the limited number of studies on this topic. This review highlights a great need for further research in female athletes, with a special focus on elite athletes. Studies should preferably implement a prospective design with adjustment for possible confounders such as stress level, sleep quality, seasonality, time until the start of an important competition and many other factors.

## Supplementary Material

Prevalence of premenstrual syndrome and premenstrual dysphoric disorder among highly trained and elite female athletes: A systematic review and meta-analysis
