# Supplementary material for: Prevalence of premenstrual syndrome and premenstrual dysphoric disorder among highly trained and elite female athletes: A systematic review and meta-analysis
Source: Biol Sport. 2025 Mar 18;42(3):211–25. doi: 10.5114/biolsport.2025.148542 (PMC12244394; doi:10.5114/biolsport.2025.148542)
Supplement: Prevalence of premenstrual syndrome and premenstrual dysphoric disorder among highly trained and elite female athletes: A systematic review and meta-analysis [file JBS-42-3-55771-s1.pdf]

SUPPLEMENTARY MATERIAL

DETAILS OF THE CRITERIA USED TO ASSESS THE QUALITY OF THE STUDIES

The maximum of total points was 10; 0 to 3 points indicated – low-quality study, 4 to 6 points – medium-quality study, and 7 to 10 points – high-quality study. Each study was assessed in terms of 3 categories: selection, comparability, and outcome. The criteria, which were taken into consideration while assessing the studies, are presented below.

SELECTION (MAXIMUM 5 POINTS)

(1) Representativeness of the sample:

(a) Truly representative of the average in the target population – all subjects or random sampling [1 point];

(b) Somewhat representative of the average in the target population – non-random sampling. We accepted studies where the sample included women from various faculties, universities, provinces etc. [1 point];

(c) Selected group of users [0 points];

(d) No description of the sampling strategy [0 points].

(2) Sample size

(a) Justified and satisfactory. We accepted every mention, e.g. calculation of sample power [1 point];

(b) Not justified [0 points].

(3) Non-respondents

(a) Response rate > 80% [1 point]

(b) Response rate < 80%, but described non-responders characteristics [1 point]

(c) Response rate < 80%, if response rate not explicitly stated [0 point]

(4) Ascertainment of the exposure (risk factor)

(a) Well defined training and performance caliber, for example 6-tiered Participant Classification Framework[1], VO_2max [2 points];

(b) No tier was given, but the % of elite athletes and the level of competition was properly described [1 point];

(c) Not properly described, or a detailed description was provided upon e-mail request, but not in the publication [0 points].

COMPARABILITY (MAXIMUM 2 POINTS)

The subjects in different outcome groups are comparable, based on the study design or analysis.

(a) Age, height and body weight were given [1 point];

(b) Inclusion and exclusion criteria clearly described [1 point];

OUTCOME (MAXIMUM 3 POINTS)

(1) Assessment of the outcome:

(a) Validated measurement tool – PMS diagnosed by Daily Symptom Record Scale, Premenstrual Symptoms Screening Tool [2 points];

(b) Non-validated measurement tool, but the tool is available or described [1 point];

(c) No description of the measurement tool [0 points].

(2) Statistical test

(a) The statistical test used to analyze the data is clearly described and appropriate, and the measurement of the association is presented, including confidence intervals and the probability level (p value) [1 point];

(b) The statistical test is not appropriate, not described or incomplete [0 points].

REFERENCES

1. Wells, G.A.; Shea, B.; O'Connell, D.; Peterson, J.; Welch, V.; Losos, M.; Tugwell, P. The Newcastl ttawa Scale (NOS) for Assessing the Quality of Nonrandomised Studies in Met nalyses Available online: http://www.ohri.ca/programs/clinical_epidemiology/oxford.asp
2. Modesti, P.A.; Reboldi, G.; Cappuccio, F.P.; Agyemang, C.; Remuzzi, G.; Rapi, S.; Perruolo, E.; Parati, G.; ESH Working Group on CV Risk in Low Resource Settings Panethnic Differences in Blood Pressure in Europe: A Systematic Review and Met nalysis. PLOS ONE 2016, 11, e0147601, doi:10.1371/journal.pone.01476

[1] McKay AKA, Stellingwerff T, Smith ES, Martin DT, Mujika I, Goosey-Tolfrey VL, Sheppard J, Burke LM. Defining Training and Performance Caliber: A Participant Classification Framework. Int J Sports Physiol Perform. 2022; 17(2):317–331. doi: 10.1123/ijspp.2021-0451

SUPPLEMENTARY TABLE

TABLE S1. Impact of premenstrual symptoms on athletic performance during training or competition

Author, year	% of female athletes			
	Not at all	Mild	Moderate	Severe
Takeda et al. 2015 [6]	55.7	31.6	8.0	4.6
Takeda et al. 2016a [4]	58.9	28.2	10.7	2.3
Takeda et al. 2016b [5]	56.5	29.5	11.5	2.5
